# Lessons from *Anaplasma phagocytophilum:* Chromatin Remodeling by Bacterial Effectors

**DOI:** 10.2174/187152612804142242

**Published:** 2012-10

**Authors:** Kristen E Rennoll-Bankert, J Stephen Dumler

**Affiliations:** Department of Pathology, The Johns Hopkins University School of Medicine, Baltimore, MD 21205, USA

**Keywords:** *Anaplasma phagocytophilum*, AnkA, *CYBB*, epigenetics, histone modifications, and NADPH oxidase

## Abstract

Bacterial pathogens can alter global host gene expression via histone modifications and chromatin remodeling in order to subvert host responses, including those involved with innate immunity, allowing for bacterial survival. *Shigella flexneri, Listeria monocytogenes, Chlamydia trachomatis*, and *Anaplasma phagocytophilum *express effector proteins that modify host histones and chromatin structure. *A. phagocytophilum* modulates granulocyte respiratory burst in part by dampening transcription of several key phagocyte oxidase genes. The *A. phagocytophilum *protein AnkA localizes to the myeloid cell nucleus where it binds AT-rich regions in the *CYBB *promoter and decreases its transcription. AT-rich regions of DNA are characteristic of matrix attachment regions (MARs) which are critical for chromatin structure and transcription. MAR-binding proteins, such as SATB1, interact with histone modifying enzymes resulting in altered gene expression. With *A. phagocytophilum* infection, histone deacetylase 1 (HDAC1) expression is increased and histone H3 acetylation is decreased at the *CYBB* promoter, suggesting a role for AnkA in altering host epigenetics and modulating gene transcription, at this, and perhaps other loci. This review will focus on how bacterial pathogens alter host epigenetics, by specifically examining *A. phagocytophilum* AnkA cis-regulation of *CYBB* transcription and epigenetic changes associated with infection.

## INTRODUCTION

Intracellular bacterial pathogens exploit host cells to promote self survival by subverting host responses, especially those related to immunity and defense. A common mechanism among bacterial pathogens is the expression of effector proteins (also known as virulence factors) that are secreted and alter host cell signaling pathways, vesicular-trafficking, and cytoskeletal pathways [1]. Alterations to the host cell eventually lead to differential gene expression and ultimately dysregulation of host cells resulting in disease. Microarray analysis of the mammalian transcriptome in the presence of intracellular pathogens reveals large subsets of genes that are similarly regulated with infection [2]. While changes in signaling pathways and transcription factors are known to alter host transcription, global changes in gene regulation are often associated with epigenetic modifications characteristic of transcriptional programs that govern cell function. 

Mammalian hosts have evolved mechanisms to detect microbial infection and to initiate protective transcriptional programs by altering chromatin structure. The best-characterized link between a bacterial stimulus and altered histones is the activation of the mitogen-activated protein kinase (MAPK) cascade. Three MAPK pathways have been defined according to the kinase that is activated: ERK pathway, JNK/SAPK pathway, and p38 kinase pathway. Lipopolysaccharide (LPS) found on the cell surface of Gram-negative bacteria is recognized by Toll-like receptor 4 (TLR4) which activates the ERK and p38 kinase pathways. Both the ERK and p38 kinases activate the effector kinases MSK1 and MSK2 that phosphorylate Ser10 on histone H3 (H3S10) [3-5]. This allows nucleosome remodeling leading to NF-κB binding and transcription of inflammatory cytokines and chemokines such as IL-12 [6, 7].

Recent investigations have provided compelling evidence that microbes also evolved mechanisms that alter mammalian chromatin structure and result in altered transcriptional programs that increase microbe fitness. The bacterial pathogens *Anaplasma phagocytophilum, Shigella flexneri, Listeria monocytogenes, *and *Chlamydia trachomatis* express effector proteins that modify host histones and chromatin structure by altering signaling pathways, interacting with histone modifying enzymes or by direct modification. This review will focus on bacterial effectors that alter host epigenetics, using *A. phagocytophilum* Ankyrin A (AnkA) nuclear translocation and the resulting changes in host cell transcription and epigenetic state as a model system.

## 1 DOES EPIGENETICS PLAY A ROLE IN *ANAPLASMA PHAGOCYTOPHILUM *INFECTION? 

### A *Anaplasma phagocytophilum* and Neutrophil Dysfunction

*Anaplasma phagocytophilum, *the causative agent of human granulocytic anaplasmosis (HGA), is a small (0.2 – 1.0 µm in diameter) Gram-negative, obligate intracellular bacterium that is transmitted to humans via tick bites [8, 9]. It has a tropism for granulocytes and their precursors, and may transiently infect endothelial cells [10, 11]. While most microbes are readily killed by the neutrophil [12], *A. phagocytophilum *is able to subvert neutrophil killing and propagate within the infected cell. How this pathogen spends its life within the hostile environment of the neutrophil is a key question this review aims to address. 

Neutrophils are the primary antimicrobial effectors in innate immunity and kill microbes using a repertoire of tools. First, neutrophils engulf microbes resulting in phagosome formation. The phagosome then acidifies and fuses with a lysosome to produce a phagolysosome. There are two general mechanisms of microbial killing once the phagolysosome has formed: oxygen-independent and oxygen-dependent. Oxygen-independent mechanisms include i) release of hydrolytic enzymes and bactericidal proteins from the neutrophil, such as elastase, bactericidal permeability-increasing protein (BPI), defensins [13], and ii) the release of neutrophil extracellular traps (NETs) by dying neutrophils [14]. Oxygen-dependent killing relies on the generation of reactive oxygen species by the nicotinamide adenine dinucleotide phosphate (NADPH) oxidase; also known as the phagocyte oxidase [13]. Upon binding and entry, *A. phagocytophilum* is able to subvert these mechanisms of killing by residing in an immature vacuole and altering neutrophil function.

In the neutrophil, *A. phagocytophilum* survives by altering key host cell functions. *A. phagocytophilum* delays spontaneous and induced apoptosis in the neutrophil, allowing the bacteria time to replicate and form morulae (>24hrs). Decreased apoptosis is the result of continued transcription of host *BCL2* family genes and stabilization of the intrinsic pathway that prevents procaspase 3 processing [15-17]. Furthermore, *A. phagocytophilum* upregulates production of chemokines, including IL-8 which is responsible for neutrophil recruitment [18]. By inhibiting its action via a blocking antibody to CXCR2, Scorpio *et al.* showed that IL-8 is important for bacterial propagation via neutrophil recruitment that results in clusters of infected cells in tissues [19]. Additionally, the bacterium alters neutrophil adhesion to endothelial cells, transmigration through endothelium, motility, degranulation, and phagocytosis. When infected with *A. phagocytophilum*, both neutrophils and HL-60 cells (a human promyelocytic cell line used to propagate *A. phagocytophilum*) shed PSGL-1 and L-selectin from their cell surface resulting in decreased adhesion to systemic and brain microvascular endothelial cells, thus preventing transmigration [20]. Selectin “shedding” occurs when infected cells degranulate, causing the release of an EDTA-inhibitable sheddase (metalloprotease), β2-integrins, CD66b, and other inflammatory components such as matrix metalloproteases [20, 21]. Overall, this leads to increased neutrophil concentrations in peripheral blood with longer life spans. 

How *A. phagocytophilum* is able to alter the neutrophil phenotype so dramatically is still an unanswered question. Transcriptional analyses have identified many defense genes and inflammatory pathways that are regulated during infection [22-24]. Due to the limited genetic and metabolic resources of *A. phagocytophilum*, it is likely that the bacterium has developed a global mechanism for altering neutrophil gene regulation. Epigenetic changes tend to globally regulate gene expression and impact major cellular programs and processes such as cell cycle progression and cell differentiation. Dysregulation of epigenetic control mechanisms often leads to dramatic phenotypic changes, and if pathogen-controlled, could benefit microbial survival and propagation, inducing pathologic changes that cause disease. An ideal example is *A. phagocytophilum*’s ability to quench neutrophil oxidative burst.

### B Subversion of the Oxidative Burst

**Fig. (1):**
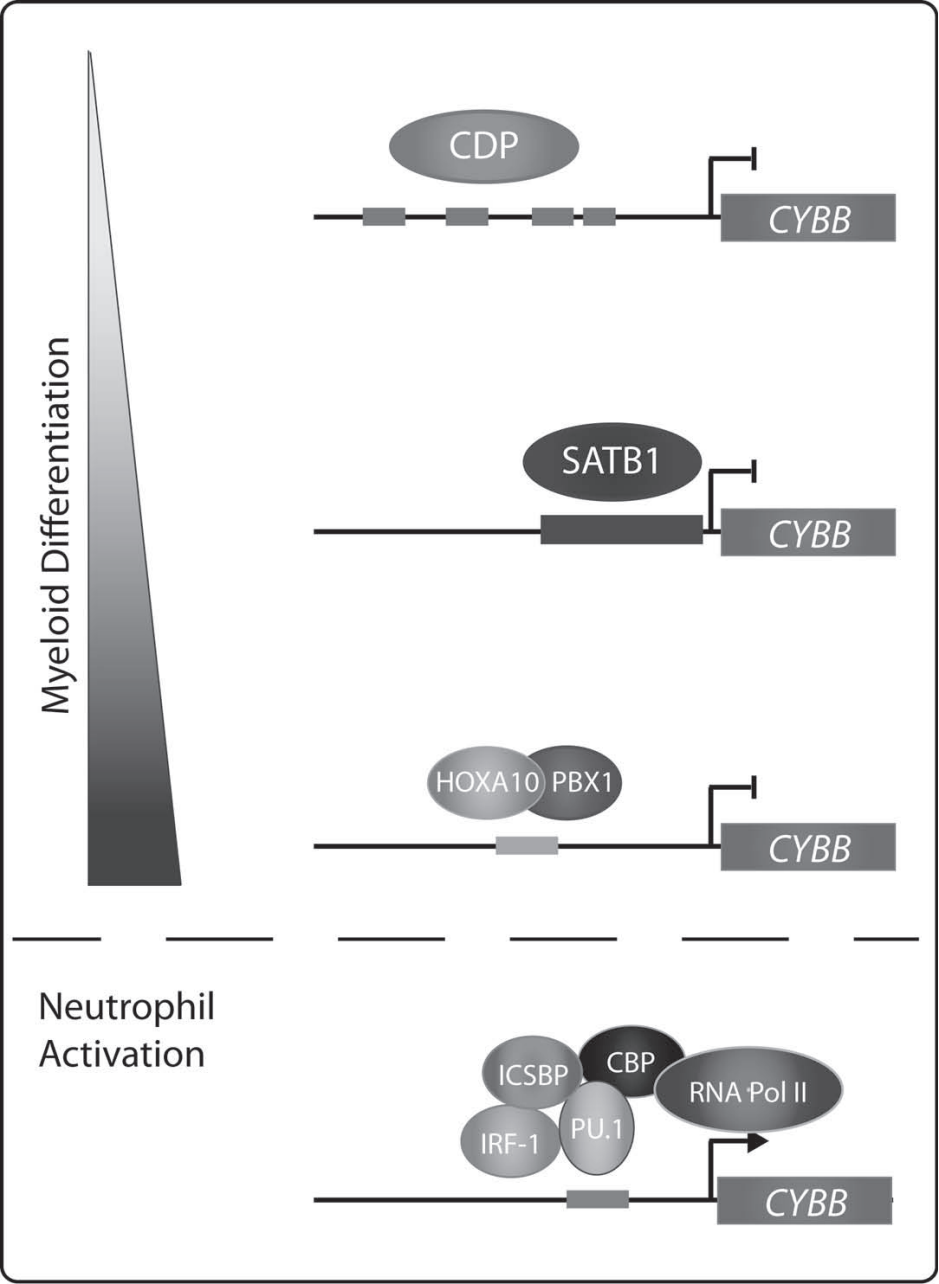
**Control of *CYBB* expression during myeloid differentiation and neutrophil activation.** Transcription factor binding within the –450 to +12 proximal region of the *CYBB* promoter is important for *CYBB* repression during myeloid differentiation and neutrophil activation in response to stimuli including IFN-γ and LPS.

Decreased production of reactive oxygen by NADPH oxidase is critical for *A. phagocytophilum *survival. When decreases in NADPH oxidase activity are prevented, *A. phagocytophilum* intracellular propagation diminishes [25]. *A. phagocytophilum *subverts NADPH oxidase killing by sequestering reactive oxygen species, inhibiting NADPH oxidase activity and decreasing expression of NADPH oxidase genes [26]. *A. phagocytophilum* does not activate NADPH oxidase assembly in the bacterial inclusions of HL-60 cells. This is in part attributed to the lack of LPS and peptidoglycan synthesis by *A. phagocytophilum *[26]. In addition, *A. phagocytophilum* decreases expression of *CYBB *and *RAC2, *two components of the NADPH oxidase, in infected neutrophils and HL-60 cells [25]. Genes encoding components of the NADPH oxidase are highly regulated during development.* CYBB* is exclusively expressed in terminally differentiated phagocytes and is induced via inflammatory agents such as IFN-γ and LPS [27]. The proximal −450 to +12 region of the *CYBB *promoter is sufficient for IFN-γ induced expression [28]. The activator PU.1 is phosphorylated by IFN-γ-activated protein kinase C resulting in PU.1 binding to the *CYBB *promoter. PU.1 in turn recruits interferon regulatory factor 1 (IRF-1) and IFN consensus-sequence binding protein (ICSBP) to form the hematopoietic associated factor-1 (HAF1) complex. The HAF1 complex then recruits CREB binding protein (CBP) resulting in the HAF1a complex which further recruits RNA polymerase II [29]. Additional activators of *CYBB *include CCAAT binding protein (CP1), binding increases during differentiation (BID)/YY1 factor, HOXA9 and IRF-2 [30-32]. In non-myeloid cells, CAATT displacement protein (CDP) inhibits *CYBB *expression by preventing activators from binding [33-35]. Committed myeloid progenitors express special AT-rich binding protein 1 (SATB-1) which represses *CYBB *transcription. Upon terminal myeloid differentiation, SATB-1 expression decreases [36, 37]. The HOXA10/PBX1 repressor binds and recruits histone deacetylase (HDAC) 2 to prevent transcription in myeloid cells until activation. Once neutrophils are activated by IFN-γ, HOXA10 is phosphorylated decreasing its ability to bind the *CYBB *promoter and allowing *CYBB *transcription (Fig. **1**) [38]. 

Downregulation of *CYBB *expression is associated with increased binding of CDP to its promoter. *A. phagocytophilum-*infected cells have increased cathepsin L activity leading to CDP cleavage and increased DNA binding [39]. CDP binding to promoters of the human neutrophil peptide 1 and C/EBPε genes, molecules important for neutrophil defense and maturation, is also increased with infection [40]. Levels of the transcription factors IRF-1 and PU.1, which are critical for *CYBB* expression, are decreased in infected HL-60 cells [39]. We identified that *A. phagocytophilum* protein AnkA binds to the *CYBB *promoter and decreases its transcription in HL-60 cells [41].

### C AnkA Binds the *CYBB* Promoter

AnkA is a 160 kDa protein with several eukaryotic motifs/domains including ankyrin repeats, a putative nuclear localization sequence and a high mobility group N chromatin-unfolding domain (HMGN-CHUD), among others [42]. The ankyrin repeat is a 33-residue motif that occurs in tandem arrays and is common among nuclear proteins that bind transcription factors. The repeats cooperatively fold into structures that allow for protein-protein interactions [43]. The HMGN-CHUD motif is found in proteins that bind nucleosomes and change chromatin structure resulting in altered transcription [44]. AnkA is secreted via a type IV secretion system and translocates to the nucleus of infected myeloid lineage cells including neutrophils [42, 45]. 

Upon translocation to the nucleus, AnkA binds AT-rich heterochromatic DNA that lacks conserved binding sequences [46]. Using chromatin immunoprecipitation, we showed that AnkA binds to the *CYBB *proximal promoter. Fine mapping of AnkA binding to the *CYBB *promoter using electrophoretic mobility shift assay (EMSA), showed that AnkA binds 2 specific promoter regions: -48 to +12 and -138 to -109 bp. Remarkably, AnkA binding correlates with predicted matrix attachment regions (MARs) in the *CYBB* promoter [41]. MARs are regions of DNA responsible for binding nuclear matrix proteins that define chromatin domains. MARs contain base unpairing regions (BURs), AT-rich genomic sequences that allow DNA to easily unwind [47, 48]. *CYBB* promoter AnkA binding regions that are mutated by replacing adenosine and thymidine with cytosine and guanine nucleotides (to disrupt BURs) have abrogated AnkA binding [41], suggesting that AnkA is a MAR binding protein. SATB-1, another MAR binding protein, also binds the *CYBB* promoter and represses *CYBB* expression by altering chromatin structure [36, 37].

**Fig. (2):**
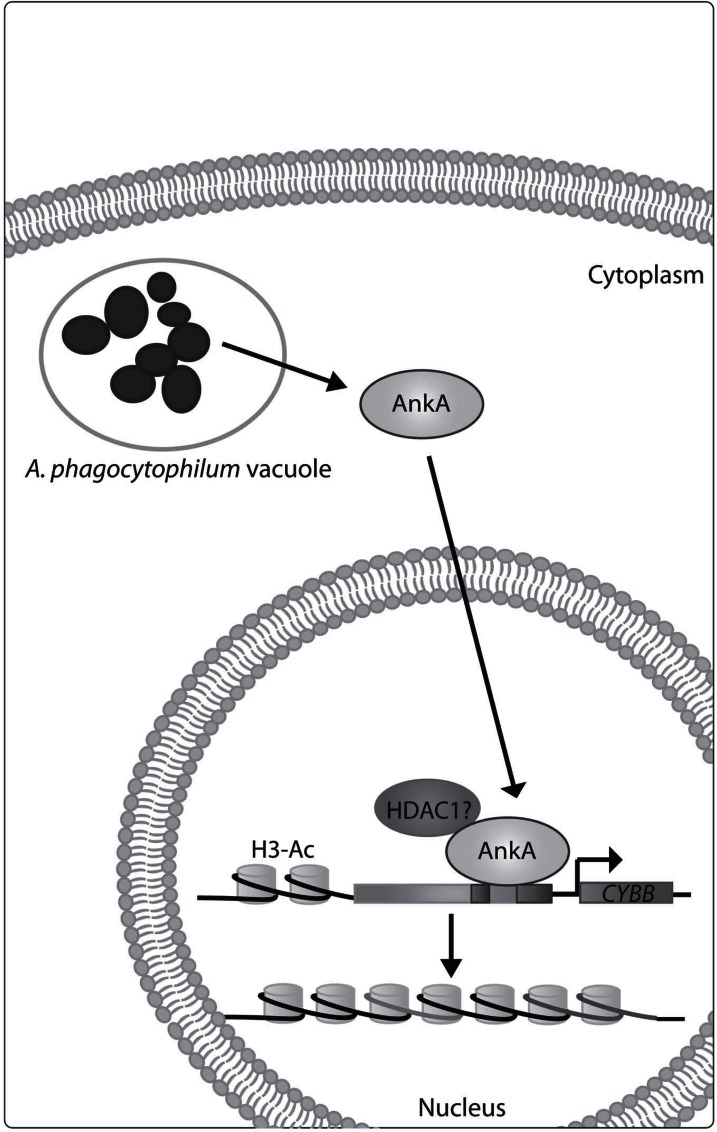
**Predicted model for AnkA control of the *CYBB* promoter.** AnkA is secreted by *A. phagocytophilum* and translocates to the nucleus of infected cells. AnkA binds AT-rich regions of the *CYBB* promoter and decreases its expression. It is predicted that AnkA decreases *CYBB* transcription by recruiting HDAC1 to decrease histone H3 acetylation and induce formation of heterochromatic DNA.

In HL-60 cells, infection with *A. phagocytophilum *results in AnkA translocation into the nucleus leading to decreased *CYBB* transcription. Moreover, cells transfected with a plasmid expressing AnkA or with recombinant AnkA also have decreased *CYBB *expression, showing that AnkA directly alters *CYBB *transcription. In addition to AnkA binding, histone H3 acetylation is decreased at the *CYBB* promoter with *A. phagocytophilum* infection, suggesting that AnkA mediates epigenetic changes at the *CYBB *promoter (Fig. **2**) [41]. 

### D The Role of Epigenetic Change with Infection

AnkA expression decreases transcription of several defense genes including* RAC2, MPO, BPI, *and* MYC.* Furthermore, infection of the THP-1 cell line (a human acute monocytic leukemia cell line with myelomonocytic features) with *A. phagocytophilum* leads to decreased expression at defense gene clusters. A decrease in H3 acetylation with *A. phagocytophilum* infection is observed in 9 of 11 defense gene promoters [49]. Together, these data suggest *A. phagocytophilum* globally down-regulates host defense genes, perhaps as a mechanism for survival. 

In addition to changes in histone marks, *A. phagocytophilum* increases expression and activity of HDAC1 and HDAC2. In THP-1 cells treated with trichostatin A (TSA) or sodium butyrate, HDAC inhibitors, *A. phagocytophilum*’s ability to propagate within the neutrophil is suppressed. In cells treated with *HDAC1* siRNA, *A. phagocytophilum *no longer blocks defense gene expression nor propagates. Silencing of *HDAC2* expression alone has no affect on *A. phagocytophilum* growth [49]. When taken together, these data suggest that *A. phagocytophilum* decreases expression of host defense genes by increasing *HDAC1* expression and activity. However, how HDAC1 is recruited to these promoters is still unknown. AnkA binds to promoters in regions found to be deacetylated at H3 with infection. Could AnkA bound to defense gene promoters be responsible for recruitment of HDAC1 and altered chromatin structure? These are important questions considering AnkA’s similarity to the MAR binding protein, SATB-1, which is known to interact with HDAC1 and alter chromatin structure. Interestingly, if AnkA is found to bind defense gene promoters, and to recruit HDAC1, thereby decreasing expression of defense genes, it would in part explain why HDAC1 expression and activity are indispensible for *A. phagocytophilum* propagation. Studies from other laboratories also describe bacterial effectors that translocate to the nucleus of host cells. These include a recent report of p200, an ankyrin repeat protein of *Ehrlichia chaffeensis*, a species related to *A. phagocytophilum*, which binds *Alu-Sx *elements in DNA [50]. It is tempting to speculate that these effectors will also affect host chromatin using similar mechanisms we describe for AnkA in *A. phagocytophilum* infection*.*

## 2 EPIGENETIC ALTERATIONS IN OTHER BACTERIA: A COMMON DENOMINATOR?

### A Shigella flexneri OspF

**Fig. (3):**
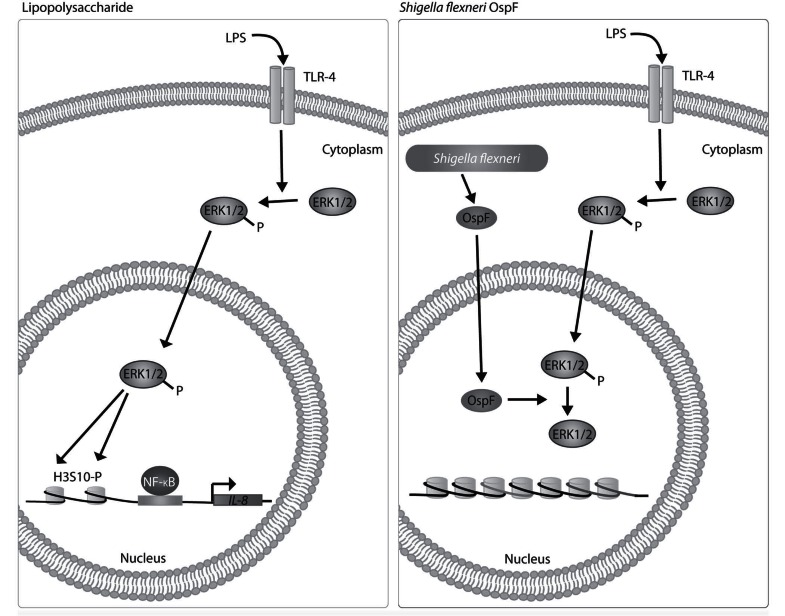
**OspF dephosphorylates MAPKs in the nucleus.** Activation of TLR-4 by LPS results in activation of MAPKs which induce phosphorylation of H3S10 allowing for nucleosome remodeling, NF-κB binding and transcription of inflammatory cytokines and chemokines such as IL-8. OspF is secreted by the *Shigella flexneri* type III secretion system and translocates to nuclei of infected cells. In the nucleus, OspF irreversibly dephosphorylates MAPKs including ERK1/2 and p38 resulting in decreased H3S10 phosphorylation and transcription of inflammatory cytokines and chemokines.

*Shigella flexneri* is a Gram-negative bacterium that infects colonic epithelium causing bacillary dysentery. Epithelial cells are critical for sensing bacterial infection by signaling the immune system via cytokines and chemokines to initiate the inflammatory response [51]. To circumvent this, *S. flexneri* has developed strategies to modify expression of proinflammatory genes within epithelial cells. LPS induces nucleosome remodeling via phosphorylation of H3S10. *S. flexneri *OspF, a type III secretion system effector, localizes to the nucleus of infected cells [52]. Through its phosphothreonine lyase activity, OspF irreversibly dephosphorylates ERK1/2 and p38 MAPKs in the nucleus thereby preventing phosphorylation of H3S10. This inhibits nucleosome rearrangement at specific MAPK target genes preventing NF-κB binding and activation of gene transcription. Specifically, OspF inhibits expression of a small set of immune genes including immediate early genes and a subset of NF-κB-responsive genes including IL-8, which is responsible for recruiting neutrophils (Fig. **3**) [53, 54]. This corroborates the finding that OspF inhibits neutrophil recruitment [52].

### B *Listeria monocytogenes* LLO and LntA

The facultative intracellular pathogen, *Listeria monocytogenes* is well adapted to survive in an extracellular location. However, depending on host environmental factors, it will also infect, survive, and replicate within the cytoplasm of macrophages and nonprofessional phagocytic cells. *L. monocytogenes* is the causative agent of listeriosis, a food-borne infection that can lead to sepsis and meningitis. Interferons released by infected cells alert neighboring cells to activate defense mechanisms including expression of IFN-stimulated genes. Interferons often lead to rapid clearance of bacterial cells with infection, yet some bacteria survive to cause severe disease, as with *L. monocytogenes *[55]. While immune defense genes are strongly activated in *L. monocytogenes*-infected macrophages *in vitro* and *in vivo*, some subsets are down-regulated early during infection [56, 57]. Two *L. monocytogenes* toxins, listeriolysin O (LLO) and *Listeria* nuclear targeted protein A (LntA), have been identified to globally alter host transcription during the early and late stages of infection via histone modifications and recruitment of histone modifying complexes [58, 59].

**Fig. (4):**
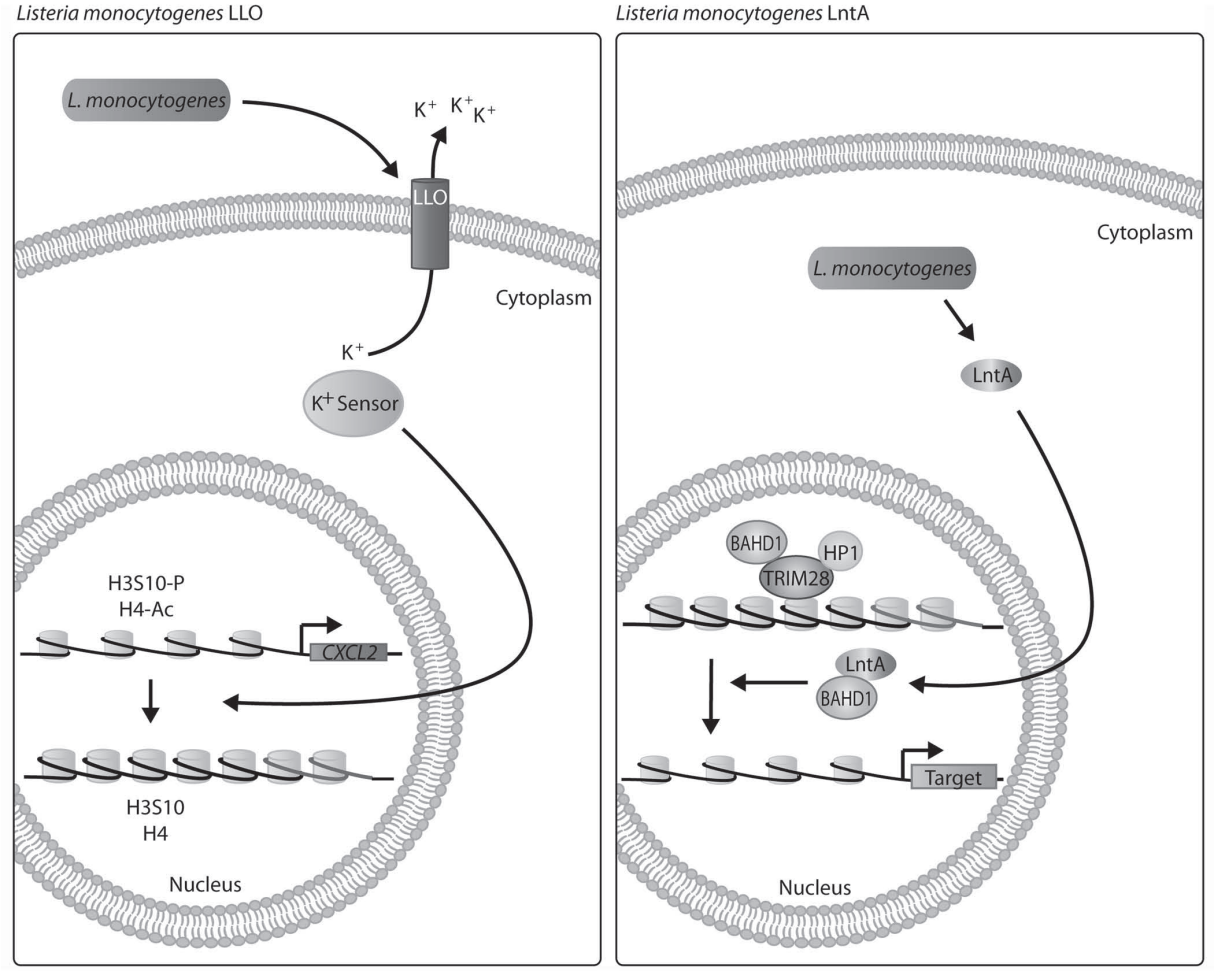
**Schematic of LLO and LntA impact on host chromatin.** Early in *L. monocytogenes* infection, LLO inserts into macrophage cell membrane and induces potassium efflux which leads to histone dephosphorylation and deacetylation. A potassium sensor such as HDAC8 could be responsible for altering the histones. During late infection, LntA is predicted to bind BAHD1 and prevent its repression of IFN-stimulated genes.

LLO is a member of the large pore-forming toxin family known as cholesterol-dependent cytolysins (CDCs), all produced by Gram-positive bacteria [60, 61]. Besides its pore-forming function, LLO is secreted into host cells before bacterial entry and acts as a signaling molecule. LLO induces MAPK and calcium signaling in a pore-forming-dependent manner and NF-κB signaling in a pore-forming-independent manner [62-64]. Additionally, LLO induces K^+^ efflux ultimately leading to dephosphorylation of H3S10 and deacetylation of H4, resulting in decreased expression of LLO-target genes such as* CXCL2* (Fig. **4**) [65]. Down-regulation of *CXCL2 *reduces recruitment of neutrophils, key effectors in the innate immune response against *L. monocytogenes *[66]. The ability of LLO to alter histone structure is reliant on LLO binding to host cells and forming pores in host cell membranes [58]. The mechanism by which K^+ ^efflux results in dephosphorylation and deacetylation is not fully understood. However, deacetylases are known to be sensitive to changes in K^+^ concentrations including histone deacetylase 8 (HDAC8) [67]. Other members of the CDC family dephosphorylate H3S10, including PFO (*Clostridium perfringens*) and PLY (*Streptococcus pneumonia*) [58].

LntA, another *L. monocytogenes* toxin, is a 205–amino acid basic protein with an N-terminal signal peptide expressed in pathogenic *L. monocytogenes. *LntA localizes to heterochromatic DNA in the nucleus where it binds BAHD1. Early in disease IFN-stimulated genes are repressed by a protein complex comprising of BAHD1, HP1, TRIM28 (or KAP-1) and HDAC 1 and 2, which promotes formation of heterochromatin. Late in disease, IFN-stimulated genes are no longer repressed by the BADH1 complex, allowing an immune response to be triggered [59, 68]. LntA prevents BAHD1 binding to DNA, thus decreasing BAHD1 repression of interferon IFN genes (Fig. **4**). Interestingly, mice infected with strains of *L. monocytogenes* that constitutively express or lack LntA have decreased bacterial burden. This suggests that tight control of LntA expression is critical for *L. monocytogenes* survival with LntA being expressed only during late infection [59].

### C *Chlamydia trachomatis* NUE

**Fig. (5):**
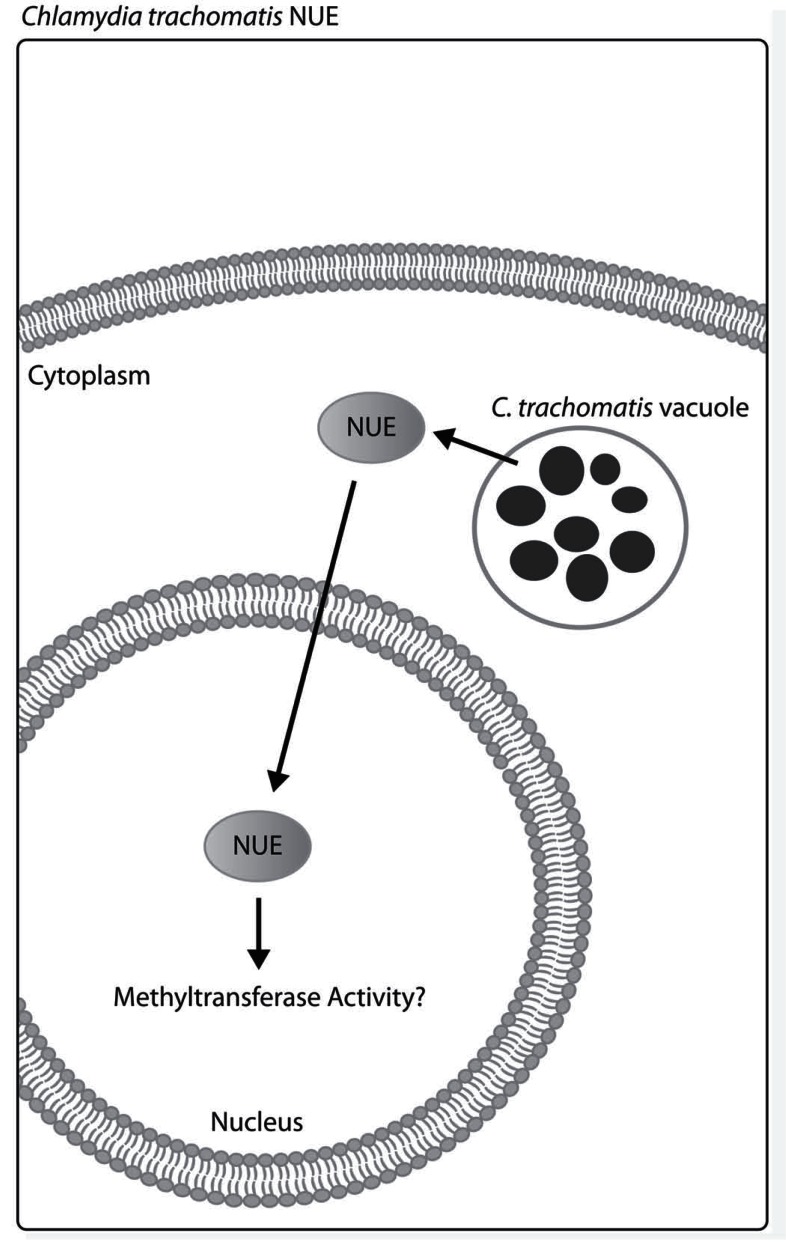
***C. trachomatis* NUE localizes to the nucleus.** NUE has methyltransferase activity which is predicted to alter chromatin structure leading to differential gene transcription upon NUE translocation into the nucleus. The gene targets of NUE have yet to be determined.

*Chlamydia trachomatis* is the most prevalent sexually transmitted bacterial pathogen. This obligate intracellular and Gram-negative bacterium most often targets epithelial cells. Encoding a type III secretion system, *C. trachomatis* secretes several effectors that have consequences for the host. Recently described by Pennini *et al.*, a SET domain-containing histone methyltransferase called nuclear effector (NUE) is secreted into the cytoplasm for translocation into the nucleus of infected epithelial cells (Fig. **5**). *In vitro* methyltransferase assays reveal that NUE methylates histones H2B, H3 and H4. Genomic analysis of other *Chlamydia *species also revealed ORFs with homology to NUE. While target genes of NUE have yet to be identified [69], this is the first known bacterial nuclear effector with the ability to directly alter mammalian histones.

## CONCLUSIONS

The study of host epigenetic changes in response to bacterial effectors is essential to better understand host-pathogen interactions. Here, we discuss several bacterial effector proteins that alter host histones and chromatin structure and that lead to subversion of the host immune system. The mechanisms by which these effectors alter histone marks need to be further clarified. Additionally, these emerging investigations raise other considerations, such as: do other bacteria express homologous effectors that result in host epigenetic changes or whether there are other global mechanisms by which bacteria alter host epigenetics still to be discovered? Recently, Mombelli *et al.* inhibited HDAC activity in macrophages and observed increased survival of *Escherichia coli *and* Staphylococcus aureus* due to impaired macrophage phagocytosis and decreased NADPH oxidase activity [70]*.* This demonstrates the importance of HDAC activity in regulating gene expression that inevitably leads to clearance of bacterial pathogens. The answers to these and other questions will be important as we continue to investigate genetic and epigenetic mechanisms of host response and subversion of these responses by bacterial pathogens.
